# Sustainable Polyurea Greases Based on Epoxidized Soybean Oil: Influence of Ureido Structure on Performance

**DOI:** 10.3390/molecules31091484

**Published:** 2026-04-29

**Authors:** Yifan Chen, Xiaoling Yao, Hongjiang Yu, Gaobo Lou

**Affiliations:** 1 College of Chemistry and Materials Engineering, Zhejiang A&F University, Hangzhou 311300, China; yfchen@zafu.edu.cn (Y.C.); 13151320460@163.com (H.Y.); 2 Zhejiang Key Laboratory of Green and Low-Carbon Utilization Technology of Agriculture and Forestry Biomass, Hangzhou 311300, China; 3 Huzhou Wanma Polymer Materials Co., Ltd., Huzhou 311300, China; yaoxiaoling2026@163.com

**Keywords:** greases, polyurea, epoxidized soybean oil, rheology, tribology

## Abstract

In this study, sustainable polyurea greases were prepared using epoxidized soybean oil (ESO) as bio-based base oil, with octadecylamine (ODA) reacted with three diisocyanates: 4,4′-diphenylmethane diisocyanate (MDI), 1,6-hexamethylene diisocyanate (HDI), and toluene diisocyanate (TDI). The diisocyanate structure dominated the thickener microstructure: MDI-ODA formed a compact short-rod fibrillar network with strong hydrogen bonding and π–π stacking, endowing the grease with the highest consistency (256), dropping point (262 °C), lowest oil separation (2.7%), and optimal thermal stability (T5% = 278 °C). HDI-ODA showed a lamellar structure with moderate performance, while TDI-ODA presented a loose porous network. Rheological tests confirmed MDI-ODA/ESO possessed the highest yield stress and structural recovery (79.5%). Tribological tests showed MDI-ODA/ESO delivered the lowest friction coefficient and wear scar diameter. Compared with non-epoxidized soybean oil (SO), ESO significantly enhanced grease performance. This improvement is attributed to the ring-opening reaction between the N–H of the ureido group and the epoxy groups of ESO, which improves thickener–oil compatibility. In addition, the polar epoxy groups promote the formation of stable lubricating films. This work verifies that diisocyanate structure and base oil epoxidation are critical for high-performance sustainable polyurea greases.

## 1. Introduction

With the advancement of industrialization, mechanical systems are increasingly required to operate under harsh and fluctuating service conditions—such as extreme temperatures, sandstorms, salt-spray atmospheres, and acidic or alkaline environments [1]. These factors inevitably aggravate friction, wear, and corrosion, thereby exerting a profound detrimental impact on equipment performance and operational longevity. Among these failure mechanisms, friction-induced wear remains the predominant cause of component degradation and energy dissipation [2,3]. It has been reported that friction accounts for approximately one-third to one-half of global energy consumption, and over 80% of mechanical failures are directly attributed to friction and wear, resulting in an economic loss equivalent to nearly 2–7% of the global GDP [4]. The application of appropriate lubricants is essential for effectively mitigating friction and wear between contacting interfaces, ultimately prolonging the service life of mechanical equipment [5,6,7,8].

Grease, a semi-solid lubricant, typically consists of a base oil (65–95%), a thickener (5–35%), and functional additives (0–10%) [9,10]. The base oil provides the primary lubricating function, while the thickener forms a three-dimensional structural network capable of entrapping the oil, and the additives impart specific performance characteristics [11]. Compared with liquid lubricants, greases not only reduce friction and wear but also offer additional advantages, including excellent resistance to leakage, a broad operating temperature range, vibration damping and noise reduction capabilities, and improved corrosion protection [12]. Owing to these merits, grease has been widely applied in various tribological components such as bearings, gears, and chains [13,14,15].

Currently, a wide variety of lubricating greases are available, among which soap-based systems—such as barium [16,17], calcium [18,19], and lithium [20,21] soaps—remain the dominant class. In contrast, non-soap greases have developed rapidly in recent years; polyurea greases [22,23,24,25] and organoclay-thickened greases [26,27] are receiving increasing attention. Among these, polyurea grease has emerged as one of the most promising non-soap alternatives. The material was first identified in 1954 by Swaken and co-workers [28] during their investigation of thickeners designed to enhance the thermal and oxidative stability of silicone oils. Owing to its excellent thermal resistance, superior colloidal stability, and inherent anti-corrosion properties, polyurea grease has been widely adopted in sectors such as steel processing, automotive engineering, electrical equipment, and defense applications [29]. It is now regarded as a key class of high-performance grease and is often used as an indicator of technological advancement in a nation’s grease manufacturing industry [30,31].

In polyurea grease, the lone-pair electrons on nitrogen increase the polarity of the urea moieties, thereby enhancing their affinity for metal surfaces [32]. Polyurea thickeners are typically formed through the addition reaction between diisocyanates and organic amines. Consequently, the molecular architectures of the reactants play a decisive role in shaping the microstructure of the resulting polyurea network, which ultimately governs the macroscopic performance of the grease. Maksimova et al. [33] examined the relationships among thickener composition, rheological behavior, and anti-wear properties by varying both the base-oil type and the amine constituents in polyurea systems. Their results showed that incorporating 25 wt% dodecyl-terminated diurea into dioctyl sebacate significantly improved the colloidal stability and yield stress of the grease. These findings highlight that the compatibility between the thickener and the base oil is a key determinant of overall performance, and that structural differences among amines can substantially influence the behavior of polyurea greases. Similarly, Ren et al. [34] synthesized polyurea greases using mineral oil while tuning the reaction between methylene diphenyl diisocyanate (MDI) and various organic amines. They reported that greases derived from MDI/cyclohexylamine or MDI/p-toluidine combinations exhibited superior physicochemical and tribological properties. To date, most studies have focused on tailoring amine structures to probe structure–property relationships in polyurea thickeners [35], whereas the influence of diisocyanate chemistry has received comparatively limited attention. The molecular structure of diisocyanate is equally crucial in determining polyurea chain architecture and, in turn, the performance of polyurea greases; systematic investigations in this area remain scarce.

In addition, the choice of base oil exerts a pronounced influence on the properties of polyurea greases. Garshin et al. [36] investigated how base-oil composition affects the low-temperature performance of polyurea greases and found that oils containing a balanced mixture of paraffinic, naphtha, and minor aromatic components achieve a compromise between low-temperature flow and structural stability. Greases formulated with these base oils exhibited moderate apparent viscosities (2136–2909 Pa·s) at −50 °C, dropping points above 240 °C, and volatilities below 15%. In another study, Wang et al. [37] prepared three polyurea greases using the same preformed thickener but different base oils (PAO40, AN30, and OSP320). Their results demonstrated that, compared with highly polar OSP320 and the less polar AN30, the nonpolar PAO40 favors the formation of a densely interconnected three-dimensional fibrous network. Low-field NMR and molecular simulation further revealed that this network more effectively traps the base oil and suppresses its mobility, leading to enhanced structural strength and improved lubrication and low-noise performance. Although mineral oils remain the most common base oils in current polyurea-grease research, they originate from non-renewable petrochemical sources and offer limited environmental compatibility [38]. This underscores the urgent need to develop renewable, bio-based oils—particularly those derived from plant feedstocks—to enable more sustainable and environmentally benign polyurea-grease formulations [39].

In addition, vegetable oils have attracted growing interest as renewable and biodegradable alternatives to mineral-based base oils for grease formulations. Their inherent advantages include high viscosity index, low volatility, excellent lubricity, and low toxicity [40]. However, unmodified vegetable oils suffer from several critical drawbacks, such as poor oxidative stability, limited hydrolytic resistance, and inadequate low-temperature fluidity, which severely restrict their direct application in lubricating greases [41]. Moreover, the relatively low polarity of natural triglycerides often leads to weak interactions with polar thickener networks, resulting in inferior colloidal stability and oil separation. To overcome these limitations, chemical modification strategies—such as epoxidation, transesterification, or estolide formation—have been developed to introduce polar functional groups (e.g., epoxy, hydroxyl, or ester groups) that enhance both the oxidative stability and compatibility with thickeners [42]. Among these, epoxidation has proven particularly effective: the introduced epoxy groups not only improve the polarity and surface affinity of the oil but also provide reactive sites for covalent or strong hydrogen-bonding interactions with functional groups in the thickener (e.g., N–H of ureido in polyurea systems). Consequently, epoxidized vegetable oils (ESO) represent a promising platform for developing high-performance, sustainable polyurea greases.

Based on the above background, this study employs epoxidized soybean oil (ESO) as a bio-based base oil and synthesizes a series of polyurea thickeners from octadecylamine (ODA) and diisocyanates (MDI, HDI, and TDI) with distinct molecular structures. The resulting greases are used to elucidate how variations in diisocyanate structure affect their physicochemical characteristics, rheological behavior, and tribological performance. Furthermore, by comparing the grease-forming capability of soybean oil before and after epoxidation, the work highlights the performance enhancements imparted by epoxidized plant oils in polyurea systems. Surface and interfacial analytical techniques were also employed to investigate the underlying frictional mechanisms. This work revealed for the first time how diisocyanate architecture governs thickener morphology and performance. Moreover, it provides direct evidence that epoxidation enables a ring-opening reaction between ureido N–H and epoxy groups, which dramatically improves thickener–oil compatibility and tribological properties beyond simple oil replacement.

## 2. Results

### 2.1. Characterization and Physicochemical Properties

**Figure 1:**
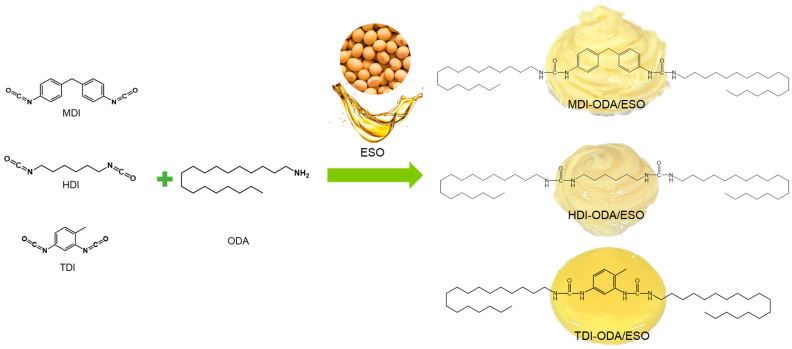
Schematic diagram of the synthesis of polyurea using different isocyanates and octadecylamine.

The synthesis process of the three types of polyurea lubricating grease is shown in Figure 1. The physicochemical properties of the three polyurea greases are closely related to the molecular structure of polyurea, and different molecular structures can significantly influence their overall performance. The consistency characterizes the hardness or softness of the grease and is closely associated with the interaction between the base oil and the thickener; stronger interactions facilitate the formation of a dense and stable three-dimensional network, thereby enhancing the mechanical stability and load-bearing capacity [43]. The dropping point serves as an important indicator of the thermal resistance of grease, reflecting the ability of the thickener network to absorb and retain the base oil. A higher dropping point indicates better structural stability of the grease under elevated temperatures, reducing the risk of softening or leakage [44]. Oil separation indicates the capability of grease to release base oil under high temperature, high pressure, or long-term storage conditions [45]. Appropriate oil separation ensures a continuous lubricating oil film at the friction interface, thereby reducing friction and wear, while excessive oil separation may lead to structural degradation and lubrication failure [46]. Therefore, rationally controlling the dropping point, consistency, and oil separation is essential for achieving long-term stable lubrication of polyurea grease under complex operating conditions [47].

**Figure 2:**
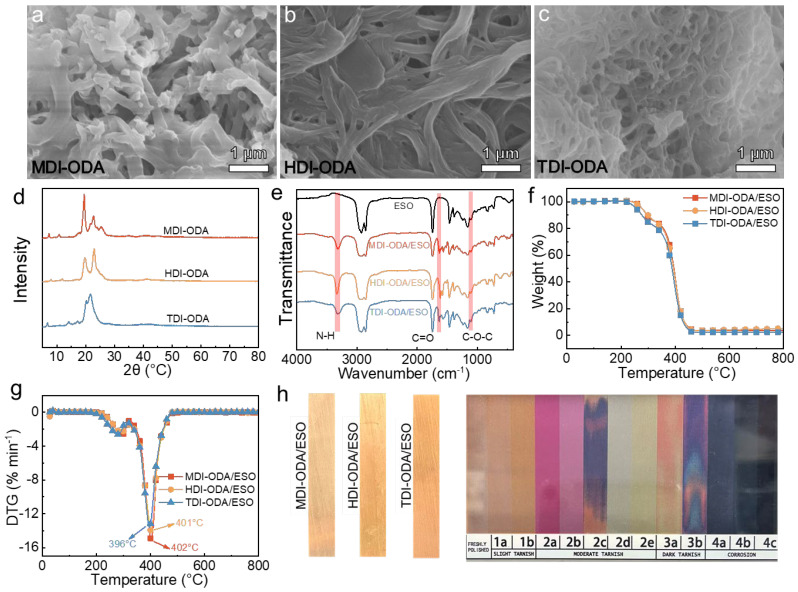
The SEM images of polyurea thickeners of (**a**) MDI-ODA, (**b**) HDI-ODA and (**c**) TDI-ODA, the (**d**) XRD and (**e**) FTIR spectra of polyurea thickeners, the (**f**) TG and (**g**) DTG curves of the polyurea greases, and (**h**) copper corrosion test of polyurea greases.

**Figure 3:**
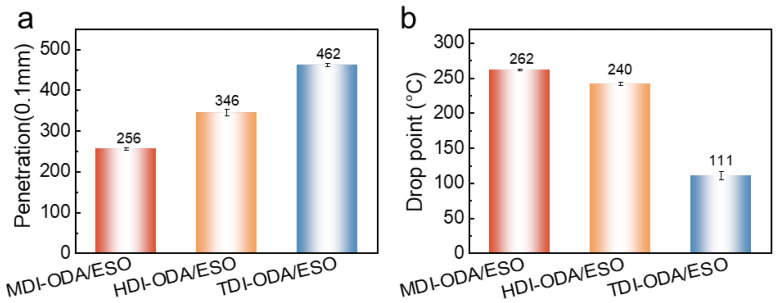
The (**a**) penetration and (**b**) drop point of polyurea.

Figure 2a–c shows the microstructures of different polyurea thickeners. As shown in Figure 2a, the MDI-ODA exhibits a compact morphology with small pore size enables the thickener network to absorb and immobilize a large amount of base oil, forming a stable three-dimensional colloidal framework and thereby endowing the grease with excellent physicochemical properties. As a result (Figure 3 and Table 1), the MDI-ODA/ESO shows the highest consistency (256), dropping point (262 °C) and the lowest oil separation (2.7%), suggesting a strong interaction between the thickener and base oil. The HDI-ODA grease presents a lamellar, sheet-like crystalline structure with interlaced stacking and moderate interlayer spacing, as shown in Figure 2b. Although such a layered morphology can also construct a three-dimensional network, the relatively weak interlayer forces (mainly van der Waals interactions and limited hydrogen bonding) and reduced contact area between particles significantly weaken the oil-holding capacity. Consequently, the HDI-ODA/ESO exhibits a lower dropping point (240 °C), reduced consistency (346), and the highest oil separation (8.7%) due to the easier oil migration along the lamellar interfaces. The TDI-ODA/ESO grease displays a loose and porous fibrous or irregularly entangled network with abundant voids and poor connectivity, which fails to build a stable gel skeleton (Figure 2c). The porous network of TDI-ODA arises from steric hindrance caused by the ortho-methyl group on the TDI molecule, which disrupts regular chain extension and favors branched or cyclic oligomers. This contrasts with the symmetric MDI (linear growth, π–π stacking) and flexible HDI (lamellar packing). XRD data confirm the disordered nature of TDI-ODA. As a result, its structural stability is markedly reduced, showing the lowest dropping point (111 °C) and the highest penetration (462). In addition, the grease becomes too fluid to meet the requirements for oil separation measurement.

Figure 2d displays the XRD patterns of three polyurea thickeners. All samples exhibit a broad and low diffuse peak (amorphous halo) around 2θ ≈ 20°, which is typically attributed to the short-range ordered structure formed by hydrogen bonding between polyurea segments. Among them, the MDI-ODA sample shows the sharpest diffraction peak, indicating the most regular structure and the densest polyurea network; the HDI-ODA sample is intermediate; and the TDI-ODA sample displays the broadest peak, suggesting that the polyurea segments in this system exist predominantly in an amorphous state with a low degree of short-range order from hydrogen bonding, which is consistent with the physicochemical property results (penetration, dropping point, oil separation) of the grease.

Figure 2e shows the FTIR spectra of the three polyurea greases. A distinct absorption peak appears at 3321 cm^−1^, which is associated with the N–H vibration of the ureido group [22]. The characteristic peaks of the carbonyl bonds (C=O) are observed at 1630 [48]. The appearance of these signals indicates the successful formation of polyurea thickener. In addition, compared with neat ESO, the polyurea greases show a clear increase in the C–O–C stretching band at ~1100 cm^−1^ (Figure 2e), indicating ring-opening of epoxy groups.

Figure 2f,g displays the decomposition profiles of the as-prepared polyurea greases to evaluate their thermal stability, and the relevant data are summarized in Table 2. The thermal decomposition temperatures (T_5%_) of the three polyurea greases show slight differences, following the order of MDI-ODA/ESO (278 °C) > HDI-ODA/ESO (276 °C) > TDI-ODA/ESO (255 °C). This result clearly demonstrates that MDI-ODA/ESO possesses the highest initial decomposition temperature, indicating superior thermal stability among the three formulations. The enhanced thermal resistance of MDI-ODA/ESO can be attributed to its densely packed, short-rod-like fibrillar network structure (Figure 2a), which arises from the rigid aromatic rings in methylenediphenyl diisocyanate (MDI). This compact architecture facilitates stronger intermolecular interactions, such as hydrogen bonding and π–π stacking, thereby improving the structural integrity of the polyurea network under elevated temperatures. Despite these differences in the initial decomposition stage, all three greases display similar maximum decomposition temperatures (approximately 400 °C), suggesting that the overall thermal degradation behavior at higher temperatures is comparable. This is because the base oil accounts for the majority (80%) of the grease and dominates the thermal degradation behavior.

For the copper corrosion test, the copper strips coated with the three polyurea greases exhibited almost no visible change compared with the polished copper. The corrosion morphologies of the copper strips after exposure to the three polyurea greases are shown in Figure 2h. The corrosion degree was rated as 1a according to the color standard chart, indicating that all three prepared polyurea greases possess excellent corrosion resistance.

**Figure 4:**
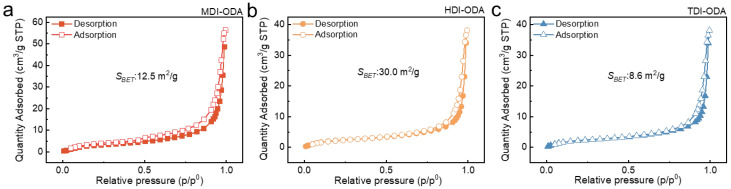
N_2_ adsorption–desorption isotherm of (**a**) MDI-ODA, (**b**) HDI-ODA and (**c**) TDI-ODA.

Figure 4 depicts the N_2_ adsorption–desorption curves of the three polyurea thickeners. The specific surface areas (SSA) of the three polyurea thickeners were measured as 12.5 m^2^/g for MDI-ODA, 30.0 m^2^/g for HDI-ODA, and 8.6 m^2^/g for TDI-ODA. Interestingly, the SSA does not exhibit a simple linear correlation with the macroscopic grease properties. Although HDI-ODA possesses the highest SSA (30.0 m^2^/g), its grease (HDI-ODA/ESO) shows only moderate consistency (346), a lower dropping point (240 °C), and a high oil separation (8.7%). This can be attributed to its lamellar, sheet-like crystalline morphology (Figure 2b), where the large SSA arises from extensive interlayer surfaces, but the weak interlayer forces (mainly van der Waals interactions) fail to form a stable three-dimensional network capable of effectively retaining the base oil. In contrast, MDI-ODA, with a moderate SSA of 12.5 m^2^/g, exhibits the best overall performance: the highest consistency (256), the highest dropping point (262 °C), and the lowest oil separation (2.7%). Its short-rod-like, densely packed fibrillar structure (Figure 2a) maximizes inter-fiber hydrogen bonding and π–π stacking, creating a robust and compact network despite a smaller surface area. TDI-ODA has the lowest SSA (8.6 m^2^/g) and forms a loose, porous, irregularly entangled network (Figure 2c), resulting in the poorest properties (penetration 462, dropping point 111 °C). Therefore, while a high SSA may contribute to oil absorption, the overall physicochemical performance is governed more by the morphological arrangement and intermolecular interactions (e.g., hydrogen bonding, aromatic stacking) than by the SSA value alone. A dense and well-connected network (MDI-ODA) with moderate SSA is superior to a high-SSA but weakly stacked lamellar structure (HDI-ODA) for achieving high thermal resistance, mechanical strength, and oil retention in polyurea greases.

### 2.2. Rheological Properties

**Figure 5:**
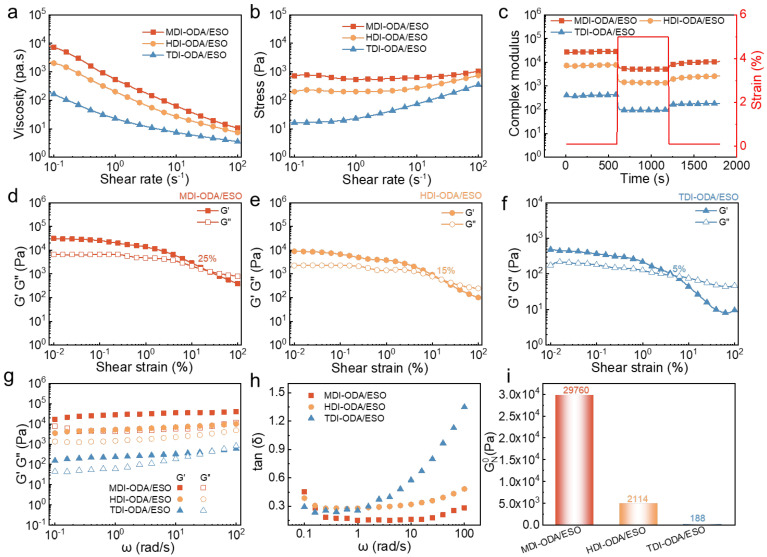
The variation in (**a**) viscosity and (**b**) stress with shear rates for polyurea greases, (**c**) structural recovery for polyurea greases, strain sweep of (**d**) MDI-ODA/ESO, (**e**) HDI-ODA/ESO and (**f**) TDI-ODA/ESO, evolution of (**g**) G′, G″ and (**h**) tan δ of polyurea greases versus frequency, (**i**) $G_{N}^{O}$ of polyurea greases.

Figure 5a presents the variation in apparent viscosity (η) with shear rate (γ) of three polyurea greases. As the shear rate increases, the viscosities of all three polyurea grease samples decrease, indicating a typical shear-thinning behavior [49]. The viscosity order can be summarized as: MDI-ODA/ESO > HDI-ODA/ESO > TDI-ODA/ESO, Figure 5b shows the shear stress (τ) as a function of shear rate (γ). At the start-up of shear, the shear stress gradually increases until it reaches the yield stress. Yield stress refers to the minimum shear stress required to initiate flow. Below this critical value, the internal network structure remains intact, and the material exhibits solid-like elastic behavior. Once the applied stress exceeds the yield stress, the internal structure is disrupted and undergoes irreversible rearrangement, leading to fluid-like behavior [50,51]. After yielding, the shear stress maintains a relatively stable state [52]. The Herschel–Bulkley rheological model (1) is used to fit the Stress-Shear rate (τ-γ) curves to obtain the rheological parameter:

(1) $$\tau =\tau_{y}+\Phi \gamma^{n}$$

where τ, τ_y_, Φ, γ, and n represent the shear stress, yield stress, plastic viscosity, shear rate and rheological index, respectively. Table 3 shows the rheological parameters of greases. It can be observed that the order of yield stress among the three polyurea greases is as follows: MDI-ODA (534 Pa)/ESO > HDI-ODA (149 Pa)/ESO > TDI-ODA/ESO (12 Pa). This result suggests that the MDI-ODA/ESO grease, with its short rod-like compact microstructure, possesses the most stable internal network structure.

Figure 5c shows the variation in the complex modulus (G*) of the three polyurea greases as a function of time. G* is an important rheological parameter that characterizes the viscoelastic behavior of greases, reflecting the combined contributions of storage and loss moduli, and can be calculated according to Equation (2) [53]. As observed, when the applied shear strain exceeds the linear viscoelastic region, the internal structure of the grease is disrupted, leading to a significant decrease in G*. Once the strain returns to the linear viscoelastic region, the structural network gradually reorganizes and recovers, resulting in an increase in G*. The degree of structural destruction and recovery during this process can be quantitatively evaluated using Equations (3) and (4) [54].

(2) $$G*=\sqrt{{G\prime }^{2}+{G\prime \prime }^{2}}$$

(3) $$Destruction=\frac{G_{0}^{*}-G_{1}^{*}}{G_{0}^{*}}\times 100$$

(4) $$Recovery=\frac{G_{2}^{*}-G_{1}^{*}}{G_{0}^{*}-G_{1}^{*}}\times 100$$

where *G*_0_* is the initial complex modulus value within the LVR; *G*_1_* is the second complex modulus value without LVR; and *G*_2_* is the complex modulus value within the LVR again. Table 4 displays the degree of recovery and destruction of samples estimated from Equations.

As summarized in Table 4, the MDI-ODA/ESO grease exhibits a destruction rate of 15.5% and a recovery rate of 79.5%. The HDI-ODA/ESO grease shows a slightly lower destruction rate of 11.0% but a comparable recovery rate of 79.0%. In contrast, the TDI-ODA/ESO grease displays the highest destruction rate (24.8%) and the lowest recovery rate (73.7%), indicating the poorest structural stability. The moderate destruction rate of MDI-ODA/ESO, together with its highest recovery rate, suggests that its short-rod-like, densely packed fibrillar network can effectively dissipate shear energy while maintaining a strong ability to re-establish hydrogen bonding and π–π stacking interactions after strain removal. Although HDI-ODA/ESO shows the lowest destruction rate, its lamellar sheet-like structure relies mainly on weak van der Waals forces, which limits its overall network cohesion and results in a slightly lower recovery compared to MDI-ODA/ESO. The high destruction and low recovery of TDI-ODA/ESO are consistent with its loose, porous, and irregularly entangled network, which lacks sufficient intermolecular interactions to resist shear-induced disruption and to rebuild the original gel skeleton. Therefore, the combination of moderate destruction resistance and superior recovery capability makes the MDI-based polyurea grease the most structurally resilient formulation, capable of withstanding repeated shear cycles in practical applications.

Figure 5d–f shows the viscoelastic behavior of the three polyurea greases under oscillatory mode. The storage modulus (G′) is a measure of the elastic energy stored by the material under external force, whereas the loss modulus (G′′) reflects the energy dissipated as heat after deformation [55]. At low shear strain, all the polyurea greases remain in the linear viscoelastic (LVE) region where G′ and G′′ are approximately parallel, indicating that the internal structure formed by the thickener network is well preserved without significant damage. As the shear strain increases, the internal structure is gradually disrupted, leading to a sharp decrease in both G′ and G′′ until they intersect (G′ = G′′), which is defined as the transition point from the solid-like to the liquid-like state and represents the critical structural strength resisting deformation [56]. The MDI-ODA/ESO grease exhibits the highest phase transition point (shear strain 25%), suggesting that the compactly stacked rod-like structure provides the highest hardness and deformation resistance. The phase transition point of HDI-ODA/ESO grease (15%) ranks second, implying that the layered crystalline structure endows the material with relatively high structural strength. In contrast, the loose and porous fibrous structure of TDI-ODA/ESO leads to the lowest structural strength, indicating the poorest shear stability among the samples.

Figure 5g–i displays the results of SAOS test of three polyurea in the LVE region. The evolution of G′ and G′′ with frequency reflects the physical entanglement and structural response characteristics of the internal three-dimensional network of the grease system [57]. The G′ is higher than the G′′ for all the polyurea greases, indicating the typical soft solid-like gel behavior of the grease, which means that the elastic response dominates over the entire frequency range [58]. From Figure 5g, the G′ gradually increases with the angular frequency (ω), suggesting that the structural support ability of the system is enhanced under high-frequency conditions, while the G′′ shows a minimum value at intermediate frequencies, implying that the energy dissipation of the grease is minimized and the structure is most stable in this region [59]. It is noteworthy that the order of G′ and G′′ with angular frequency is consistent with the variation trend of yield stress, indicating a significant correlation between the macroscopic mechanical properties and the microscopic structural response. Figure 5h shows the evolution of tan δ with ω, where a plateau region appears in the intermediate frequency range, revealing the dynamic equilibrium state of the grease structure at this stage. The plateau modulus ($G_{N}^{O}$) is defined as the G′ at the minimum tan δ, which can be used to characterize the density of physical entanglement in the colloidal network [60]. The results show that the MDI-ODA/ESO grease exhibits the highest $G_{N}^{O}$ (29,760 Pa), indicating that the compact rod-like packed structure forms the densest entanglement network, thereby endowing the grease with excellent structural stability and mechanical properties.

### 2.3. Tribological Properties

**Figure 6:**
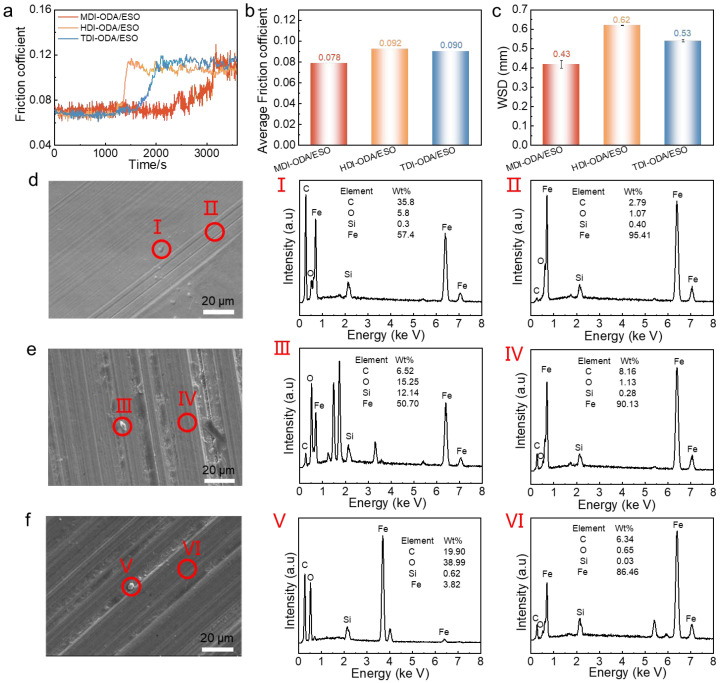
The (**a**) friction coefficient curves, (**b**) average friction coefficient and (**c**) wear scar diameter of polyurea greases, the SEM images and EDS results of wear scar for (**d**) MDI-ODA/ESO, (**e**) HDI-ODA/ESO and (**f**) TDI-ODA/ESO. I–VI correspond to the positions within the red circles in the SEM image.

Figure 6 presents the tribological performance of the greases measured by a four-ball tester. Due to the dynamic changes in lubrication states, the COFs fluctuate over time (Figure 6a). MDI-ODA/ESO exhibits the lowest friction coefficient and the smallest wear scar diameter, followed by HO/ESO and TO/ESO (Figure 6b,c). This performance is closely related to their yield stress and structural strength. The higher yield stress of MDI-ODA/ESO indicates that its three-dimensional polyurea network is denser and more stable, resisting structural collapse under shear and effectively retaining the base oil to form a continuous and stable lubricating film. The integrity of the oil film is a key factor in friction behavior. Greases with lower structural strength are more prone to network collapse under shear, leading to increased oil bleeding and disrupted film continuity, resulting in so-called “oil starvation”. Local disruption of the lubricating film causes direct contact between rough surfaces, stress concentration, and accelerated wear, which increases both the friction coefficient and the wear scar diameter. Additionally, the rigid aromatic rings in MDI promote stronger hydrogen bonding and π–π stacking between polyurea chains, which enhances the mechanical strength and load-bearing capacity of the network and suppresses oil migration and separation. In additions, A certain amounts of abrasive particles can be observed on the wear scar surfaces lubricated by the three kinds of polyurea greases (Figure 6d–f). The EDS was employed to analyze the elementary composition of the worn surfaces and abrasive debris. The oxygen content of the abrasive debris is higher than that on the worn surface, indicating that oxidative wear occurred on the contact interface. The formation of the oxide layer may result from the localized high temperature and surface activation reactions, which provide a buffering effect against direct metal contact, but also indicate the rupture of the lubricating film and the occurrence of a boundary lubrication state [34]. Overall, the MDI-ODA/ESO system effectively suppresses the formation of abrasive particles and the occurrence of oxidative wear, thereby significantly improving its anti-wear performance.

### 2.4. Performance Comparison Between Epoxidized Soybean Oil (ESO) and Un-Epoxidized Soybean Oil (SO) as Base Oils for Polyurea Grease

**Figure 7:**
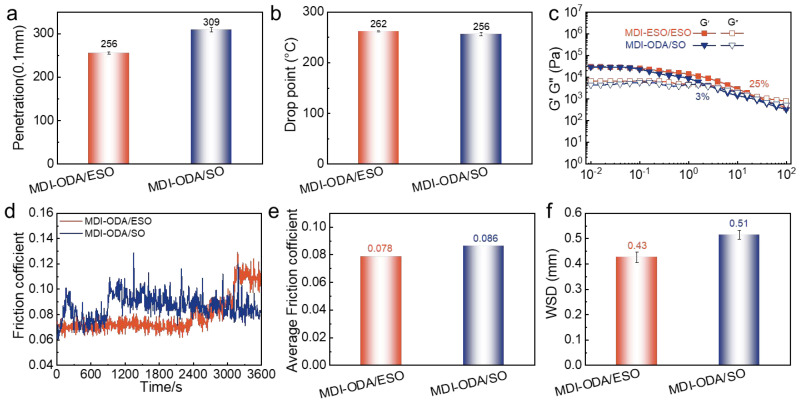
The performance comparison between MDI-ODA/ESO and MDI-ODA/SO: (**a**) Penetration, (**b**) drop point, (**c**) strain sweep, (**d**) friction coefficient curves, (**e**) average friction coefficient and (**f**) wear scar diameter.

To further demonstrate the advantages of epoxidation, a comparative grease denoted MDI-ODA/SO was prepared using un-epoxidized soybean oil (SO) as the base oil, following the same synthesis procedure and with the MDI-ODA thickener. MDI-based polyurea greases dominate the market, especially in applications requiring high thermal stability and mechanical robustness. Therefore, we selected MDI as the representative system to demonstrate the benefits of epoxidation (ESO vs. SO) because the results are most relevant to real world formulations. The physicochemical, rheological and tribological properties of MDI-ODA/SO were evaluated alongside those of MDI-ODA/ESO, and the results are summarized in Figure 7.

As shown in Figure 7a, MDI-ODA/ESO exhibits a cone penetration of 256, which is considerably lower (i.e., harder) than that of MDI-ODA/SO (309), indicating a stronger thickening effect and a more compact internal network. The dropping point of MDI-ODA/ESO (262 °C) is also higher than that of MDI-ODA/SO (256 °C), reflecting improved thermal stability (Figure 7b). In the rheological strain sweep test (Figure 7c), the flow point (the strain at which G′ = G′′) of MDI-ODA/ESO is 25%, significantly higher than the 3% observed for MDI-ODA/SO. This dramatic difference suggests that the ESO-based grease can withstand much larger deformation before the internal structure collapses, demonstrating superior mechanical stability and shear resistance. Tribological evaluations (Figure 7d–f) further confirm the superiority of the ESO. The average friction coefficient of MDI-ODA/ESO is 0.078, lower than that of MDI-ODA/SO (0.086), and the wear scar diameter (WSD) is reduced from 0.51 mm to 0.43 mm. These results clearly show that the use of ESO leads to both lower friction and better anti-wear performance.

The remarkable performance enhancement can be attributed to two synergistic factors. First, during the preparation of the polyurea grease, the active hydrogen atoms on the ureido groups (–NH–C(=O)–NH–) are capable of reacting with the epoxy rings of ESO via a ring-opening reaction. This covalent or strong polar linkage between the thickener and the base oil dramatically improves their mutual compatibility, which is absent in the non-epoxidized SO system. The enhanced compatibility results in a more homogeneous and stable three-dimensional network, where the base oil is more effectively anchored to the polyurea fibers. Consequently, the grease exhibits a harder consistency, a higher dropping point, a much larger flow strain threshold, and improved structural stability. Second, the epoxy groups themselves are polar moieties that possess excellent affinity toward metal surfaces. This favorable interaction enables ESO to form a stable and robust lubricating film on the friction interfaces, effectively reducing direct metal-to-metal contact and further enhancing the tribological performance. In contrast, the non-epoxidized SO lacks such polar functionality, resulting in weaker oil-film formation and inferior friction-reduction and anti-wear capabilities. This comparison unequivocally demonstrates that epoxidation of soybean oil is a highly effective strategy for developing high-performance sustainable polyurea greases.

## 3. Materials and Methods

### 3.1. Materials

The following materials were used: octadecylamine (ODA), 4,4′-diphenylmethane diisocyanate (MDI), 1,6-hexamethylene diisocyanate (HDI), and toluene diisocyanate (TDI). Epoxidized soybean oil (ESO) and soybean oil (SO) are also purchased from the Aladdin Reagent Co., Ltd. (Shanghai, China). All re agents were used as received without further purification. The detailed information ESO and SO as follow: Acidity (mg KOH/g): 0.45000 mg/KOH/g, Iodine value (g I_2_/100 g): ≤5 g I_2_/100 g, Epoxy value (mol/100 g, for ESO): 6.6 mol/100 g, Fatty acid composition: epoxy linoleate (51~57%), epoxy oleate (32–36%), epoxy palmitate (2.4–6.8%).

### 3.2. Preparation of Polyurea Greases

Polyurea greases were prepared via a one-step synthesis method [22]. The thickener content was controlled at 20 wt% (30 g thickener, 120 g base oil). In a typical procedure, the diisocyanate (MDI, HDI, or TDI) and octadecylamine (ODA) were each dissolved in 60 g of ESO in a 250 mL glass beaker under mechanical stirring (300 rpm, paddle stirrer) in an oil bath at 80 °C. An excess of isocyanate groups was used to ensure complete reaction of the amine groups, with a molar ratio of NCO:NH_2_ = 1.2:1. The exact masses of starting materials for each grease were as follows: MDI-ODA/ESO: MDI 10.73 g, ODA 19.27 g, ESO 120 g; HDI-ODA/ESO: HDI 8.17 g, ODA 21.83 g, ESO 120 g; TDI-ODA/ESO: TDI 8.38 g, ODA 21.62 g, ESO 120 g. After both components were completely dissolved, the two oil solutions were mixed and the reaction was continued at 80 °C for 1 h under stirring. Then, a small amount of water (0.2 g) was added to quench the excess isocyanate groups, and the temperature was raised to 100 °C for 15 min. Finally, the temperature was increased to 180 °C and held for 1 h (refining step). After cooling to room temperature, the grease was homogenized by three passes through a three-roll mill to obtain the final polyurea grease.

### 3.3. Characterization

The polyurea thickener was ahead extracted from the colloid system of grease by petroleum ether. The scanning electron microscopy (SEM, Hitachi SU 8010, Tokyo, Japan) was used to observe the morphology of thickener and wear scar. The chemical structures of polyurea grease were characterized via Fourier-transform infrared spectroscopy (FTIR, Brucker Vertex-70, Bremen, Germany). The crystalline structure of thickener was investigated using an X-ray diffractometer (XRD, Smart Lab 9, Tokyo, Japan). The thermal stability of the polyurea greases was evaluated using a TA Q500 thermogravimetric analyzer (New Castle, DE, USA) under N_2_ atmosphere by heating from 30 to 800 °C with a ramp rate of 10 ◦C/min. The specific surface areas of thickeners were evaluated by automated N_2_ gas adsorption–desorption equipment (ASAP2460, Micromeritics, Norcross, GA, USA).

### 3.4. Physicochemical Measurement

The penetration was conducted by the lubricating grease cone penetrometer (SYD-2801 C, Shanghai Changji Geological Instrument Co., Ltd., Shanghai, China) in terms of ASTM D217. The dropping point was tested by the drop point tester (WQD-1A, Shanghai INESA Physico-Optical Instrument Co., Ltd., Shanghai, China) in terms of ASTM-D566 [61]. The oil separation rate was measured by the cone net method according to NB/SH/T 0324-2010 [62]. The copper strip test followed the national standard GB/T 7326-87 [63].

### 3.5. Rheological Measurement

The rheological properties of polyurea greases were performed by the rotational rheometer (ARES-G2, TA Instruments, New Castle, DE, USA) with a parallel geometry (25 mm diameter, 1 mm gap). The dynamic viscosity test was performed in a rotation mode with the shearing rate of 0.1–100 s^−1^.The oscillation mode was used to evaluate the viscoelastic properties (by changing a shear strain of 0.01–100% and an angular frequency of 10 r/min). Small amplitude oscillatory shear (SAOS) was performed by an angular frequency of 0.1–100 rad/s and a shear strain of 0.1 Pa. The influence of strain conditions on the viscoelastic properties was elucidated through a recovery test that comprised three distinct phases: initially, a strain of 0.1% within the linear viscoelastic region was applied for a duration of 600 s; subsequently, the sample was subjected to a strain of 5% for an equivalent period; and ultimately, the conditions were replicated to match those of the initial phase.

### 3.6. Tribological Measurement

The tribological properties of the polyurea greases were tested by a four–ball tester (Hengxu SGW-10A, Jinan HengXu Testing Machine Technology Co., Ltd., Jinan, Shandong, China). For the four–ball test, the upper steel ball was mounted on the main shaft and rotated against the three same stationary steel ball with applied loads of 392 N, a rotating velocity of 1200 r/min, a test period of 3600 s. The wear scar diameter (WSD) was measured by the self-contained optical microscope with the accuracy of 0.001 mm. All the tests at the same working conditions were preformed thrice times to reduce error.

## 4. Conclusions

In this work, three polyurea greases (MDI-ODA/ESO, HDI-ODA/ESO, and TDI-ODA/ESO) were successfully synthesized using epoxidized soybean oil as a renewable base oil and diisocyanates with different molecular structures. The influence of the ureido structure on the physicochemical, rheological, and tribological properties was systematically evaluated. The main conclusions are summarized as follows:

(1) Microstructure–property relationship: The MDI-ODA thickener formed a short-rod-like, densely packed fibrillar network, which provided the most stable colloidal structure. This resulted in the highest dropping point (262 °C), greatest consistency (256), and lowest oil separation (2.7%). The HDI-ODA thickener exhibited a lamellar sheet-like structure with moderate performance, while the TDI-ODA thickener formed a loose, porous network with the poorest physicochemical properties. All three polyurea greases exhibited excellent corrosion resistance (rated 1a in copper strip tests), demonstrating their ability to protect metal surfaces under harsh conditions.
(2) Rheological performance: All three greases displayed typical shear-thinning behavior. The MDI-ODA/ESO grease exhibited the highest yield stress, highest plateau modulus (29,760 Pa), and best structural recovery rate (79.5%), indicating superior resistance to mechanical deformation and excellent thixotropic recovery. The TDI-ODA/ESO grease showed the highest destruction rate (24.8%) and the lowest recovery rate (73.7%), reflecting its weak network integrity.
(3) Tribological performance: The MDI-ODA/ESO grease achieved the lowest friction coefficient and smallest wear scar diameter, attributed to the rigid aromatic rings in MDI which promote stronger hydrogen bonding and π–π stacking between polyurea chains, and which enhances the mechanical strength and load-bearing capacity of the network and suppresses oil migration and separation.
(4) Epoxidation benefit: Compared to MDI-ODA/SO, MDI-ODA/ESO exhibited harder consistency (256 vs. 309), higher dropping point (262 vs. 256 °C), larger flow strain (25% vs. 3%), lower COF (0.078 vs. 0.086), and smaller WSD (0.43 vs. 0.51 mm). The enhancements stem from (i) the ring-opening reaction between ureido H and epoxy groups improving thickener-oil compatibility, and (ii) polar epoxy groups forming a robust lubricating film on metal surfaces.

These findings provide valuable guidance for the rational design of sustainable polyurea greases through molecular engineering of the diisocyanate structure and the use of bio-based base oils. In addition, the limitations and future directions are as follow: This study is limited to short-term laboratory evaluations without real-bearing validation, and the proposed ring-opening mechanism remains indirectly supported. Future work should include long-term stability tests, bearing rig trials, direct spectroscopic evidence (e.g., solid-state NMR), and life-cycle assessment.

## Figures and Tables

**Table 1:** The oil separation rate and copper corrosion of polyurea greases.

Samples	Oil Separation (*w*/*w*%)	Copper Corrosion
MDI-ODA/ESO	2.7	1a
HDI-ODA/ESO	8.7	1a
TDI-ODA/ESO	/	1a

**Table 2:** TG data for MDI-ODA/ESO, HDI-ODA/ESO, and TDI-ODA/ESO.

Samples	T_5%_ (°C)	T_max_ (°C)	*Y*_c_ (%)
MDI-ODA/ESO	255	402	3.8
HDI-ODA/ESO	276	401	5.2
TDI-ODA/ESO	278	396	4.9

**Table 3:** The rheological parameters of MDI-ODA/ESO, HDI-ODA/ESO, and TDI-ODA/ESO.

Samples	Yield Stress (τ_y_, Pa)	Plastic Viscosity (Φ, Pa·s)	Rheological Index (n)
MDI-ODA/ESO	534	21	0.67
HDI-ODA/ESO	149	32	0.62
TDI-ODA/ESO	12	11	0.74

**Table 4:** Structural recovery rate and damage rate of the three polyurea greases.

Samples	Destruction (%)	Recovery (%)
MDI-ODA/ESO	15.5	79.5
HDI-ODA/ESO	11.0	79.0
TDI-ODA/ESO	24.8	73.7

## Data Availability

The data presented in this study are available upon request from the corresponding author.
